# Mentoring Across Academia and Communities: A Holistic Approach Involving Needs of the Mentee

**DOI:** 10.1093/geroni/igaa057.3191

**Published:** 2020-12-16

**Authors:** Karen Kopera-Frye

**Affiliations:** New Mexico State University, Las Cruces, New Mexico, United States

## Abstract

What is mentoring? Mentoring is defined as a professional relationship in which an experienced person assists another in developing specific skills and knowledge enhancing the mentee’s professional and personal growth. Mentoring benefits include: Knowledge transfer, creating a mentoring culture, and challenging the mentee to move beyond their comfort zone. Carmel and Paul (2015) describe self-selected mentoring as a process in which a mentee identifies a potential mentor based on similarities in interests and need. Findings indicated mentees experienced opportunities in career advancement, expanded thinking, scholarly confidence, facilitation of a collaborative culture, and understanding the importance of goal setting. Mentoring as a health promotion or intervention strategy has become widespread in communities. Two mentoring approaches will be discussed: a traditional format with students and faculty in academia, another utilizing an intergenerational approach with Latinx and Native American families. Recommendations will be discussed in terms of underlying core similarities across the venues.

